# ﻿Taxonomic review of the millipede genus *Touranella* Attems, 1937, with a redescription of the type species, *T.gracilis* Attems, 1937, and descriptions of three new species from Laos (Diplopoda, Polydesmida, Paradoxosomatidae)

**DOI:** 10.3897/zookeys.1238.147550

**Published:** 2025-05-16

**Authors:** Natdanai Likhitrakarn, Sergei I. Golovatch, Khamla Inkhavilay, Somsak Panha, Chirasak Sutcharit

**Affiliations:** 1 Program of Agriculture, Faculty of Agricultural Production, Maejo University, Chiang Mai 50290, Thailand Maejo University Chiang Mai Thailand; 2 Institute of Ecology and Evolution, Russian Academy of Sciences, Leninsky pr. 33, Moscow 119071, Russia Institute of Ecology and Evolution, Russian Academy of Sciences Moscow Russia; 3 Department of Biology, Faculty of Natural Science, National University of Laos, P.O. Box 7322, Dongdok, Vientiane, Laos National University of Laos Vientiane Laos; 4 Animal Systematics Research Unit, Department of Biology, Faculty of Science, Chulalongkorn University, Bangkok 10330, Thailand Chulalongkorn University Bangkok Thailand; 5 Academy of Science, The Royal Society of Thailand, Bangkok 10300, Thailand Academy of Science, The Royal Society of Thailand Bangkok Thailand

**Keywords:** Alogolykini, Indochina, key, map, taxonomy

## Abstract

The flat-back millipede genus *Touranella* Attems, 1937 is currently comprised of 13 species, including three new ones from Laos: *T.chenla* Likhitrakarn, **sp. nov.**, *T.srisonchaii* Likhitrakarn, **sp. nov.**, and *T.jaegeri* Likhitrakarn, **sp. nov.** The generic diagnosis is updated, a key is given to all known species, and their distributions are mapped. Additionally, *T.gracilis*, the type species of *Touranella* from Vietnam, has been redescribed and illustrated based on type specimens.

## ﻿Introduction

The flat-back millipede genus *Touranella* Attems, 1937 belongs to the family Paradoxosomatidae Daday, 1889, which is among the largest in the entire class Diplopoda, with 1000+ species, 200+ genera and 22 tribes currently recognized globally (e.g., Nguyen and Sierwald 2013; Enghoff et al. 2015). Most of this outstanding diversity is confined to Indo-Australia where the Paradoxosomatidae dominate the Australian millipede fauna.

The family is presently divided into three subfamilies: Paradoxosomatinae Daday, 1889, Australiosomatinae Brölemann, 1916, and Alogolykinae Hoffman, 1963. Within the subfamily Alogolykinae there are only two tribes, Alogolykini Hoffman, 1963 and Polydrepanini Jeekel, 1968, with some seven genera in each tribe, and a total of approximately 80 species. This subfamily is much smaller in terms of generic and species diversity compared with the other subfamilies. The Alogolykinae is strictly Oriental in distribution, ranging from Pakistan, with the whole of India and Sri Lanka in the west, through the Himalayas, southern China, Myanmar and Thailand, to central and southern Vietnam in the east and southeast (Golovatch et al. 2021).

The tribe Alogolykini Hoffman, 1963 consists of seven genera: *Alogolykus* Attems, 1936 (1 species, Myanmar), *Tetracentrosternus* Pocock, 1895 (4 species, Myanmar, Thailand, and southern China), *Singhalorthomorpha* Attems, 1914 (3 species, Sri Lanka), *Yuennanina* Attems, 1936 (3 species, southern China), *Touranella* Attems, 1937 (10 species, from Nepal, Laos, and Vietnam), *Curiosoma* Golovatch, 1984 and *Carlogonopus* Golovatch, Aswathy, Bhagirathan & Sudhikumar, 2021 (each with 1 species, India) (Golovatch et al. 2021; Nguyen et al. 2023).

**Figure 1.**
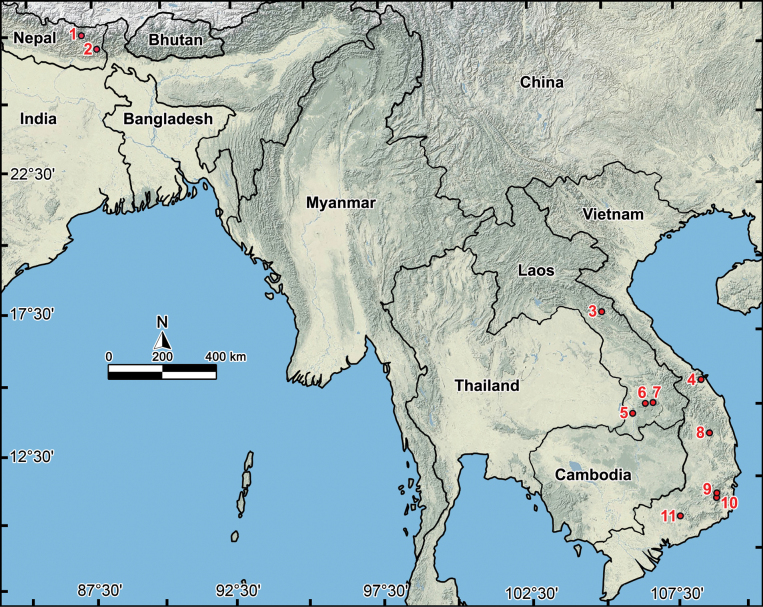
Distribution of the genus *Touranella* Attems, 1937. 1 *T.pilosa* Golovatch, 2016, 2 *Touranellahimalayaensis* Golovatch, 1994, 3 *T.jaegeri* sp. nov., 4 *T.gracilis* Attems, 1937, 5 *T.chenla* sp. nov., 6 *T.srisonchaii* sp. nov., 7 *T.champasak* Nguyen, Sierwald & Ware, 2023, 8 *T.trichosa* Golovatch & Semenyuk, 2018, 9 *T.logunovi* Golovatch, 2024, 10 *T.hirsuta* Golovatch, 2009 and *T.peculiaris* Golovatch, 2009, 11 *T.cattiensis* Golovatch & Semenyuk, 2010 and *T.moniliformis* Golovatch & Semenyuk, 2018.

*Touranella* belongs to the tribe Alogolykini and was initially described for a single, and therefore type, species, *T.gracilis* Attems, 1937. This species is characterized by a strongly reduced gonopodal femorite and a prominent, largely rod-like solenomere arising from the prefemorite. Later, a second species, *T.himalayaensis* Golovatch, 1994 was described from Nepal, highlighting a significant geographic gap of ca 2,500 km from Vietnam. Between 2009 and 2023. An additional eight species were described, of which six were found in the southern Annamite Ranges in Laos and Vietnam (Golovatch 2009a, 2009b, 2024; Golovatch and Semenyuk 2010, 2018; Nguyen et al. 2023). The sole remaining species, *T.pilosa* Golovatch, 2016, was discovered in Nepal. This has increased the overall number of *Touranella* species to ten. The present study is devoted to treating new material collected in southern Laos during several field trips, because of the discovery of three new species. In this paper we have revised the entire genus *Touranella*, providing an updated diagnosis, a revised catalogue, a key to species, and a distribution map (Fig. 1). Additionally, the type species, *T.gracilis*, has been redescribed and illustrated based on the types housed in the Natural History Museum in Vienna, Austria.

## ﻿Materials and methods

New millipede specimens were hand-collected in Laos from 2013 to 2017. All animals were euthanized in an ethical way according to the criteria set by the American Veterinary Medical Association (2020). They were then preserved in a 75% ethanol solution for further morphological examination. The Animal Care and Use Protocol Review No. 2523009 was applied. Live specimens were photographed in the laboratory using a Nikon 700D digital camera with a Nikon AF-S VR 105 mm macro lens. Subsequently, specimens were examined, measured, and photographed under a Nikon SMZ 745T trinocular stereo microscope equipped with a Canon EOS 5DS R digital SLR camera. Images were processed and edited with Adobe Photoshop CS6. Line drawings were created based on photographs and examined under the stereo microscope equipped with a digital SLR camera. For scanning electron microscopy (SEM), gonopods were coated with an 8 nm gold layer using a CCU-010 high vacuum sputter and a carbon coater (Safematic). Images were captured with a TESCAN VEGA3 scanning electron microscope operated at 5 keV acceleration voltage. Following SEM examination, gonopods were returned to 75% ethanol. The type material is deposited in the Museum of Zoology, Chulalongkorn University (**CUMZ**), Bangkok, Thailand and the Senckenberg Research Institute (**SMF**), Frankfurt, Germany.

The terminology for denoting the gonopodal and somatic structures primarily follows Golovatch (2016), Golovatch and Semenyuk (2018) and Nguyen et al. (2023). The abbreviations used for specific gonopodal structures are as follows: **d** = process d, **e** = process e, **fe** = femoral part (= femorite), **g** = lamina g, **ll** = lamina lateralis, **lm** = lamina medialis, **pfe** = prefemoral part (= prefemorite), **sl** = solenomere, **sph** = solenophore, and **u** = shoulder u.

Coordinates and elevations were recorded using Garmin GPSMAP 60 CSx and Garmin eTrex 30 devices with the WGS84 datum. The accuracy of the recorded data was subsequently verified using Google Earth Pro v. 7.3.6. In the catalogue sections, **D** stands for the original description, subsequent descriptive notes or appearance in a key, **R** for a subsequent record or records, **K** for the appearance in a key, and **M** for a mere mention.

## ﻿Taxonomy

**Family Paradoxosomatidae Daday, 1889**

**Subfamily Alogolykinae Hoffman, 1963**

**Tribe Alogolykini Hoffman, 1963**

### ﻿Genus Touranella

Taxon classificationAnimaliaPolydesmidaParadoxosomatidae

Attems, 1937

63E9E7D3-32BE-5983-A0C6-96635E26470F

Touranella Attems, 1937: 231 (D).
Touranella – Attems 1938: 233 (D); Hoffman 1963: 591 (K); 1980: 172 (M); Jeekel 1968: 64 (M); Golovatch 1994: 187 (D, M); 2009a: 6 (M, K); 2009b: 120 (M); 2016: 139 (M, K), 2024: 408 (D); Nguyen and Sierwald 2013: 1179 (M); Golovatch and Semenyuk 2010: 125 (M); 2018: 16 (M); Nguyen et al. 2023: 171 (D, K).

#### Diagnosis.

Body small to medium-sized (~ 9–25 mm long, ~ 0.7–3.3 mm wide), with 20 rings. Paraterga poorly to moderately developed. Sternal lobe or cone(s) present between ♂ coxae 4. First pair of ♂ without femoral tubercles (= adenostyles). Transverse metatergal sulci distinct. Legs with neither modifications nor adenostyles. Gonopod with a long and subcylindrical coxite, slightly curved caudally; prefemoral part (= prefemorite) short, ~ 3–4 × shorter than acropodite; femoral part (= femorite) strongly reduced or very short compared to solenophore, solenomere mostly rod-shaped or subflagelliform, sheathed distally by solenophore; both lamina medialis and lamina lateralis well developed; femoral process (fp) present or absent.

#### Type species.

*Touranellagracilis* Attems, 1937, by original designation.

#### Other species included.

*Tournaellacattiensis* Golovatch & Semenyuk, 2010, *T.champasak* Nguyen, Sierwald & Ware, 2023, *T.chenla* sp. nov., *T.jaegeri* sp. nov., *T.himalayaensis* Golovatch, 1994, *T.hirsuta* Golovatch, 2009, *T.logunovi* Golovatch, 2024, *T.moniliformis* Golovatch & Semenyuk, 2018, *T.peculiaris* Golovatch, 2009, *T.pilosa* Golovatch, 2016, *T.srisonchaii* sp. nov., and *T.trichosa* Golovatch & Semenyuk, 2018.

#### Remarks.

This genus was originally described as monotypic, based on *Touranellagracilis* Attems, 1937, which was distinguished by its markedly reduced gonopodal femoral part, the presence of a femoral process, and densely setose metaterga. The recognition of *T.himalayaensis* further supported these diagnostic features. However, subsequent discoveries of seven additional species in Vietnam have expanded the generic diagnosis, revealing further key variations such as the absence of a gonofemoral process and the presence of smooth metaterga (Golovatch 2009a, 2009b, 2016, 2024; Golovatch and Semenyuk 2010, 2018). More recently, *T.champasak* from Laos was described by Nguyen et al. (2023), which included a brief discussion of the genus and its geographic distribution. The latest contribution has described another new species from southern Vietnam (Golovatch 2024). Below a species catalogue of *Touranella* is presented, arranged in chronological order.

### ﻿ Touranella gracilis

Taxon classificationAnimaliaPolydesmidaParadoxosomatidae

Attems, 1937

AF25E355-5C7B-5FBD-AFF7-3435864ECA48

Figs 2 , 3

Touranella gracilis Attems 1937: 231 (D).
Touranella gracilis – Attems 1938: 233 (D); Jeekel 1968: 64 (M); Golovatch 1983: 182 (M); 1994: 186 (D, M); 2009a: 6 (M, K); 2009b: 120 (M); 2016: 139 (M, K); Enghoff et al. (2004): 40 (L); Golovatch and Semenyuk 2010: 125 (M); 2018: 16 (M); Nguyen and Sierwald 2013: 1180 (L); Nguyen et al. 2023: 170 (M, K).

#### Type material examined.

***Holotype*.** • ♂ (NHMW-3531), Vietnam, Danang Province, Lienchieu, 1931, leg. C. Dawydoff. ***Paratype*.** • ♀ (NHMW-3531), same locality, together with holotype.

#### Redescription.

Length 12.5 (♂) or 14.4 mm (♀), width of midbody pro- and metazona 0.73 and 0.97 mm (♂) or 1.17 and 1.47 mm (♀), respectively.

**Figure 2.**
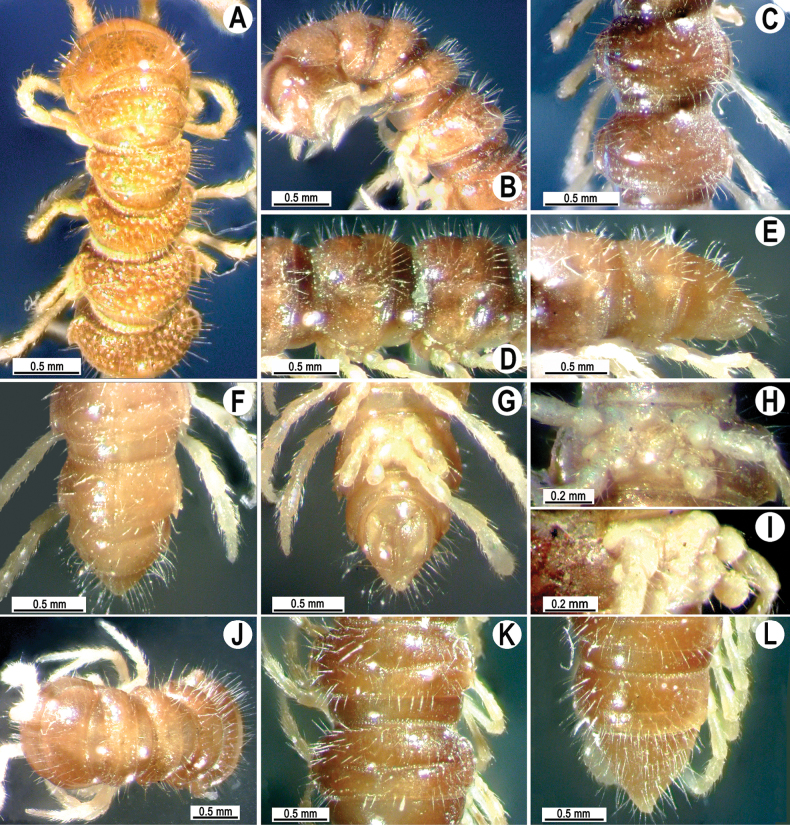
*Tournaellagracilis* Attems, 1937 **A–I** ♂ holotype **J–L** female paratype **A, B, J** anterior part of body, dorsal, lateral, and dorsal views, respectively **C, D, K** rings10 and 11, dorsal, lateral, and dorsal views, respectively **E–G, L** posterior part of body, lateral, dorsal, ventral, and dorsal views, respectively **H, I** sternal cones between coxae 4, subcaudal and sublateral views, respectively.

Coloration of alcohol material after long-term preservation dark red brown (Fig. 2A–G, J–L) with pale castaneous brown paraterga and epiproct, yellowish antennae, venter, and legs (Fig. 2A–L) (vs body color almost black with pale yellowish antennae and legs, as given in the descriptions by Attems 1937).

Clypeolabral region and vertex sparsely setose, epicranial suture distinct. Antennae rather short, reaching to body ring 3 (♂) or ring 2 (♀) when stretched dorsally. In width, ring 2 < 3 < collum < head < ring 4 < 5–16 (♂, ♀), thereafter body gently and gradually tapering towards telson. Collum with abundant, small, setiferous knobs; anterior edge broadly rounded, narrowly bordered, fused to callus; caudal corner very broadly rounded, not produced past the rear tergal margin; lateral edge with two-minute incisions, slightly declined ventrad (Fig. 2A, B, J).

Tegument smooth and shining, prozona very finely shagreened, metaterga roughly granulate-tuberculate and shagreened; surface below paraterga finely microgranulate (Fig. 2A–F, J–L). Postcollum metaterga with abundant, small, irregular, setiferous knobs. Tergal setae long, slender, ~ 1/2 length of metaterga. Axial line rather faint but traceable both on pro- and metaterga. Paraterga strongly developed (Fig. 2A–F, J–L), lying low (at ~ 1/2–1/3 midbody height); anterior edge narrowly rounded and narrowly bordered, fused to callus; caudal corner narrowly rounded to nearly pointed, not produced past the rear tergal margin; lateral edge with three evident, lateral, setigerous incisions on poreless calluses and two strong ones (anterior) on pore-bearing calluses. Calluses on paraterga delimited by a sulcus only on dorsally. Ozopores evident, lateral, each lying in an ovoid groove at ~ 1/4 in front of caudal corner. Transverse metatergal sulci usually distinct (Fig. 2A, C, F, K, L), incomplete on ring 4, complete on metaterga 5–18, line-shaped, very deep, not reaching bases of paraterga, faintly ribbed at bottom. Stricture between pro- and metazona wide, line-shaped, rather deep, evidently ribbed at bottom down to base of paraterga (Fig. 2A–F, K, L). Pleurosternal carinae with an anterior narrowly rounded crest on ring 2, thereafter missing.

Epiproct (Fig. 2E, G) conical, flattened dorsoventrally, with two-minute apical papillae; tip subtruncate; pre-apical papillae absent. Hypoproct subtriangular, setiferous knobs at caudal edge well-separated and evident (Fig. 2G).

Sterna sparsely setose, without modifications; an entire, short, linguiform, setose sternal lobe between ♂ coxae 4 (Fig. 2H, I). A paramedian pair of evident tubercles in front of gonopods aperture. Legs moderately long and slender, slightly incrassate in ♂, midbody legs ~ 1.3–1.4 (♂) or 1.0–1.1 × (♀) as long as body height, prefemora without modifications, ♂ tarsal brushes absent.

**Figure 3.**
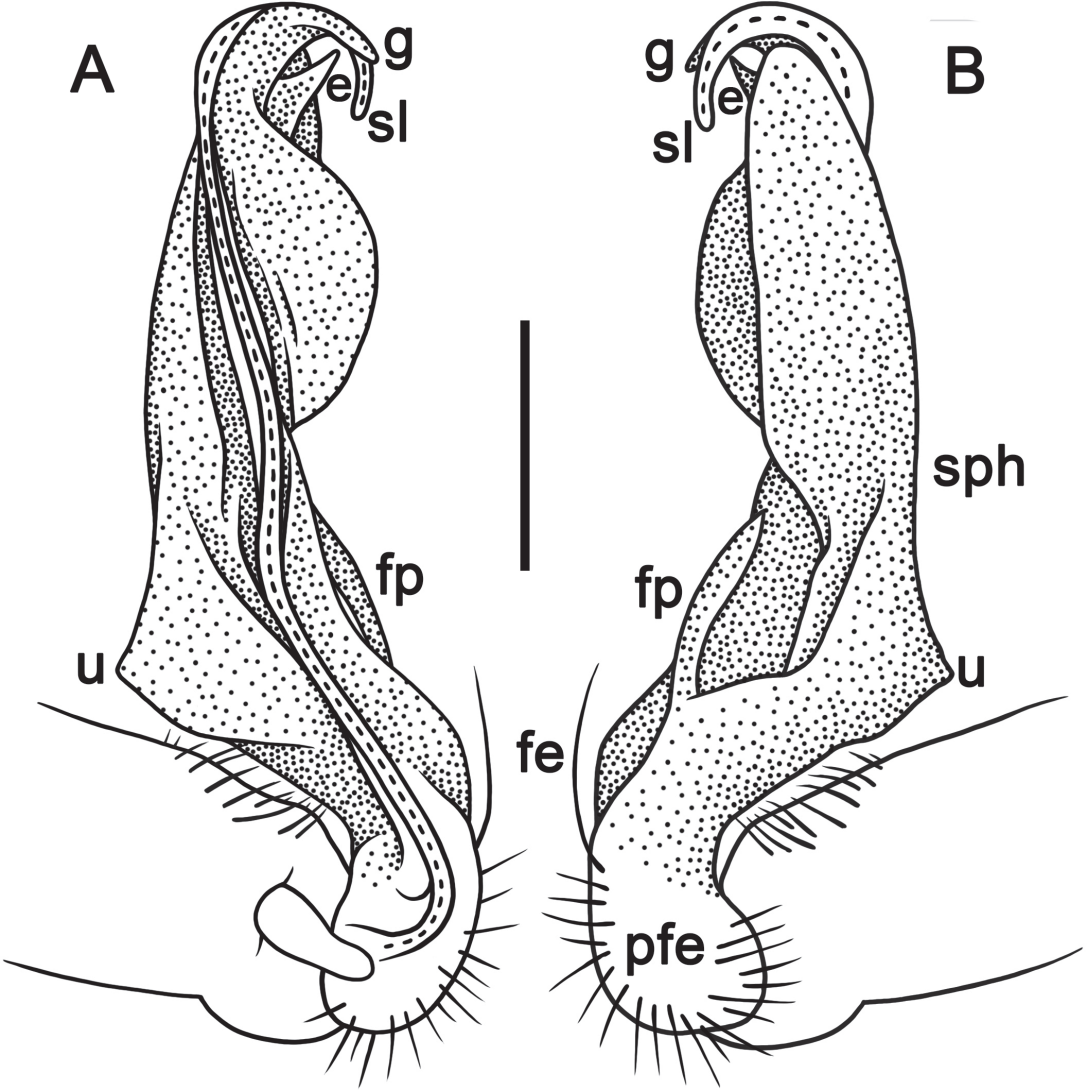
*Tournaellagracilis* Attems, 1937, ♂ holotype, left gonopod **A, B** mesal and lateral views, respectively. Abbreviations: e = process e, fe = femoral part, g = lamina g, pfe = prefemoral part, sl = solenomere, sph = solenophore, u = shoulder u. Scale bar: 0.2 mm.

Gonopods simple and stout (Fig. 3A, B); coxa slightly curving caudally, densely setose distodorsally. Prefemoral part (pfe) densely setose, ~ 1/4 the length of acropodite (femoral part + postfemoral part) (Fig. 3A, B). Femoral part (fe) strongly reduced, with a prominent, simple, lance-shaped lateral prefemoral process (fp) (Fig. 3B) and a fully medial, strong, long, flagelliform solenomere (sl) (Fig. 3A, B). Solenophore (sph) suberect, slightly twisted distally, strongly developed, sheathing most of solenomere (sl), with a lateral shoulder (u) at the base, distally bearing a pointed process (e) and a narrow unciform lamona (g) (Fig. 3A, B).

#### Distribution.

It is known only from the type locality and appears to be endemic to Vietnam.

#### Remarks.

Although Golovatch (1994) examined and illustrated the gonopodal structure of this species, nearly all other morphological features remained ignored. The present study provides a comprehensive description of the species which appears to be endemic to Vietnam.

### ﻿ Touranella himalayaensis

Taxon classificationAnimaliaPolydesmidaParadoxosomatidae

Golovatch, 1994

6833D6E6-29DD-5BE1-A70E-C7110CE07A75

Touranella himalayaensis Golovatch, 1994: 186 (D).
Touranella himalayaensis – Golovatch 2009a: 6 (M, K); 2009b: 120 (M); 2016: 139 (M, K); Golovatch and Semenyuk 2010: 125 (M); 2018: 16 (M); Nguyen and Sierwald 2013: 1180 (L); Nguyen et al. 2023: 170 (M, K).

#### Remarks.

The original description is based on specimens collected from Paniporua and Dhorpar Kharka, 2,300–2,700 m a.s.l., in mixed broadleaved or *Rhododendron*-*Lithocarpus* forests in Panchthar District, Nepal. The species is considered endemic to Nepal (Golovatch 1994).

### ﻿ Touranella peculiaris

Taxon classificationAnimaliaPolydesmidaParadoxosomatidae

Golovatch, 2009

0E4B9744-86D5-5479-97E7-C5209605ED31

Touranella peculiaris Golovatch, 2009a: 4 (D, K).
Touranella peculiaris – Golovatch 2009b: 120 (M); 2016: 132 (R); Golovatch and Semenyuk 2010: 125 (M); 2018: 16 (M); Nguyen and Sierwald 2013: 1180 (L); Golovatch 2016: 139 (M, K); Nguyen et al. 2023: 170 (M, K).

#### Remarks.

This species was first documented in Nui Ba Nature Reserve, 12°10'N, 108°40'E, 1,400–1,900 m a.s.l., near Lang Lanh, Bi Doup, Lam Dong Province, Vietnam. It is an endemic species confined to Vietnam (Golovatch 2009a, 2016).

### ﻿ Touranella hirsuta

Taxon classificationAnimaliaPolydesmidaParadoxosomatidae

Golovatch, 2009

9066EEE7-67CD-5401-A3E3-610205B03437

Touranella hirsuta Golovatch, 2009b: 119 (D, M).
Touranella hirsuta – Golovatch and Semenyuk 2010: 123 (M); 2018: 16 (M); Nguyen and Sierwald 2013: 1180 (L); Golovatch 2016: 139 (M, K), 2024: 408 (D, R); Nguyen et al. 2023: 170 (M, K).

#### Remarks.

This species was also described from Nui Ba Nature Reserve, 12°10'N, 108°40'E, 1,400–1,900 m a.s.l., near Lang Lanh, Bi Doup, Lam Dong Province, Vietnam. Apparently, the species is endemic to Vietnam (Golovatch 2009b, 2024).

### ﻿ Touranella cattiensis

Taxon classificationAnimaliaPolydesmidaParadoxosomatidae

Golovatch & Semenyuk, 2010

37F8CDDD-7224-5A50-A507-4F65203BA911

Touranella cattiensis Golovatch & Semenyuk, 2010: 123 (D).
Touranella cattiensis – Semenyuk et al. 2011: 40 (M); Golovatch et al. 2011: 81 (M); Nguyen and Sierwald 2013: 1180 (L); Golovatch 2016: 139 (M, K); Golovatch and Semenyuk 2018: 14 (M); Nguyen et al. 2023: 170 (M, K).

#### Remarks.

This species was initially documented in Nam Cat Tien National Park, 11°26'48.2"N, 107°26'26.2"E, 137 m a.s.l., inhabiting deciduous tropical forests dominated by *Dipterocarpus* species along the riverside of Dongnai River in Dongnai Province, Vietnam. Apparently, this species is endemic to Vietnam (Golovatch and Semenyuk 2010).

### ﻿ Touranella pilosa

Taxon classificationAnimaliaPolydesmidaParadoxosomatidae

Golovatch, 2016

F3D3222F-3010-5A7F-A631-1366FC47E396

Touranella pilosa Golovatch, 2016: 136 (D, K).
Touranella pilosa – Golovatch and Semenyuk 2018: 16 (M); Nguyen et al. 2023: 170 (M, K).

#### Remarks.

This species was originally described from Sankhua Sabha District, Nepal, 2,600–2,800 m a.s.l., inhabits *Quercussemecarpifolia*, *Rhododendron* scrub forests. Endemic to Nepal (Golovatch 2016).

### ﻿ Touranella moniliformis

Taxon classificationAnimaliaPolydesmidaParadoxosomatidae

Golovatch & Semenyuk, 2018

B2F1D131-B0E6-54D1-B99A-CAAD94BB3E36

Touranella moniliformis Golovatch & Semenyuk, 2018: 13 (D).
Touranella moniliformis – Nguyen et al. 2023: 170 (M, K).

#### Remarks.

The original description of the species recorded from Cat Tien National Park, 11°26'N, 107°21'E, 180 m a.s.l., within the monsoon broadleaved lowland tropical forest of Dong Nai Province, Vietnam. Apparently, endemic to Vietnam (Golovatch and Semenyuk 2018).

### ﻿ Touranella trichosa

Taxon classificationAnimaliaPolydesmidaParadoxosomatidae

Golovatch & Semenyuk, 2018

E0F02CA9-A674-5FB9-8F1E-37F52E65DDF8

Touranella trichosa Golovatch & Semenyuk, 2018: 16 (D).
Touranella trichosa – Nguyen et al. 2023: 170 (M, K).

#### Remarks.

This species was originally described from Kon Ka Kinh National Park, Gia Lai Province, Vietnam. Apparently, endemic to Vietnam (Golovatch and Semenyuk 2018).

### ﻿ Touranella champasak

Taxon classificationAnimaliaPolydesmidaParadoxosomatidae

Nguyen, Sierwald & Ware, 2023

BA312F6C-380B-50CD-988F-A029E1D45575

Touranella champasak Nguyen et al., 2023: 171 (D, K).

#### Remarks.

This species has recently been described from Ban Thongvay (= Xekatam), 15°14.288'N, 106°31.891'E, 1,095 m a.s.l., located on the Bolaven Plateau, Champasak Province, Laos. This species represented the first record of the genus *Touranella* from Laos, also reporting another species apparently endemic to Laos (Nguyen et al. 2023).

### ﻿ Touranella logunovi

Taxon classificationAnimaliaPolydesmidaParadoxosomatidae

Golovatch, 2024

44F8C92B-EC5E-5414-86DC-964EB5C3C948

Touranella logunovi Golovatch, 2024: 408 (D).

#### Remarks.

This species was described from Lam Dong Province, Durong District, Bidoup – Nui Ba National Park, near field station, 12°10'38.47"N, 108°40'49.28"E, 1450–1500 m a.s.l., mid-montane mixed tropical forest. Apparently, the species is endemic to Vietnam (Golovatch 2024).

### ﻿ Touranella chenla

Taxon classificationAnimaliaPolydesmidaParadoxosomatidae

Likhitrakarn sp. nov.

97B290B7-A139-5D3E-BDE6-D5AA21F7EFF7

https://zoobank.org/AC32C0D3-FFF1-4F01-ABE9-D3955EA8FA39

Figs 4 , 5 , 6

#### Type material.

***Holotype*** • ♂ (CUMZ-PD0031), Laos, Champasak Province, Pakse District, Vat Phou, 107 m, 14°50'52.9"N, 105°49'38.8"E, 23.7.2013, leg. Chirasak Sutcharit.

#### Diagnosis.

**Figure 4.**
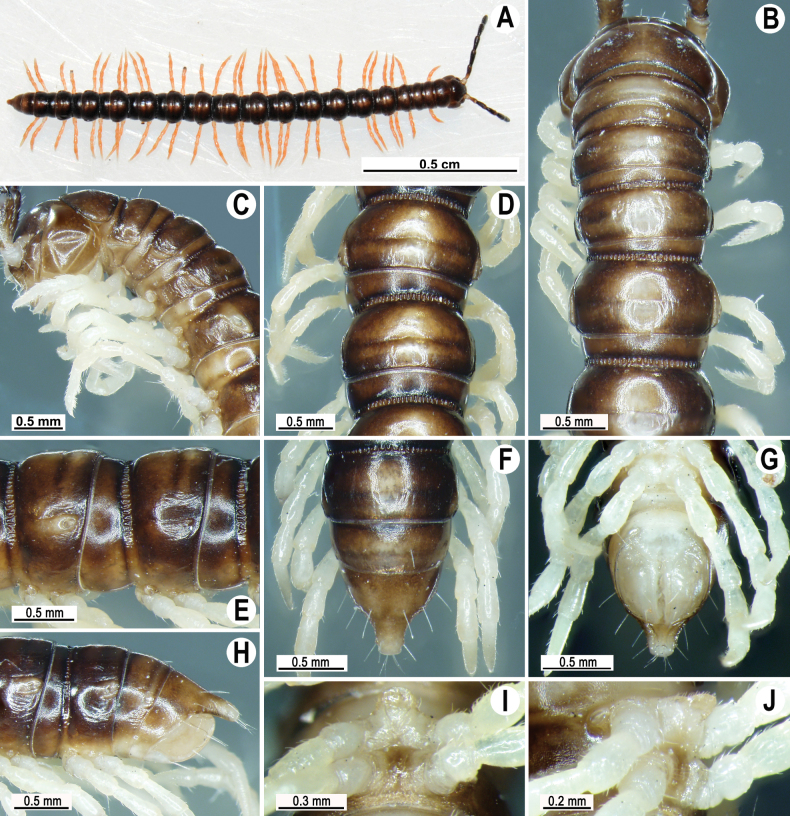
*Touranellachenla* sp. nov., ♂ holotype **A** habitus, live coloration **B, C** anterior part of body, dorsal and lateral views, respectively **D, E** rings 10 and 11, dorsal and lateral views, respectively **F–H** posterior part of body, dorsal, ventral, and lateral views, respectively **I, J** sternal cones between coxae 4, subcaudal and sublateral views, respectively.

**Figure 5.**
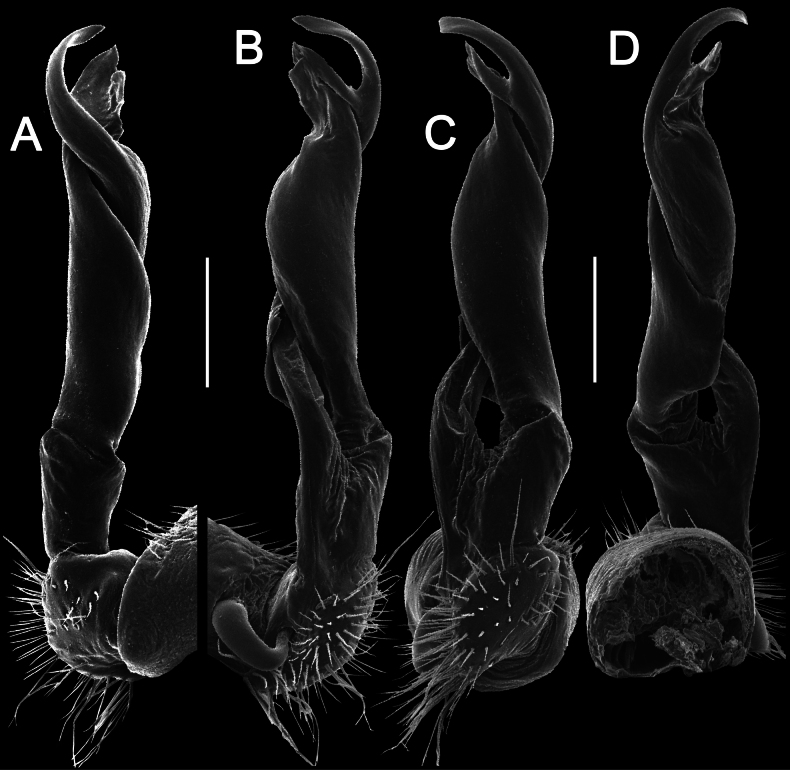
*Touranellachenla* sp. nov., ♂ holotype, right gonopod **A, B** lateral and mesal views, respectively. Abbreviations: d = process d, fe = femoral part, ll = lamina lateralis, lm = lamina medialis, pfe = prefemoral part, sl = solenomere, sph = solenophore. Scale bar: 0.2 mm.

**Figure 6.**
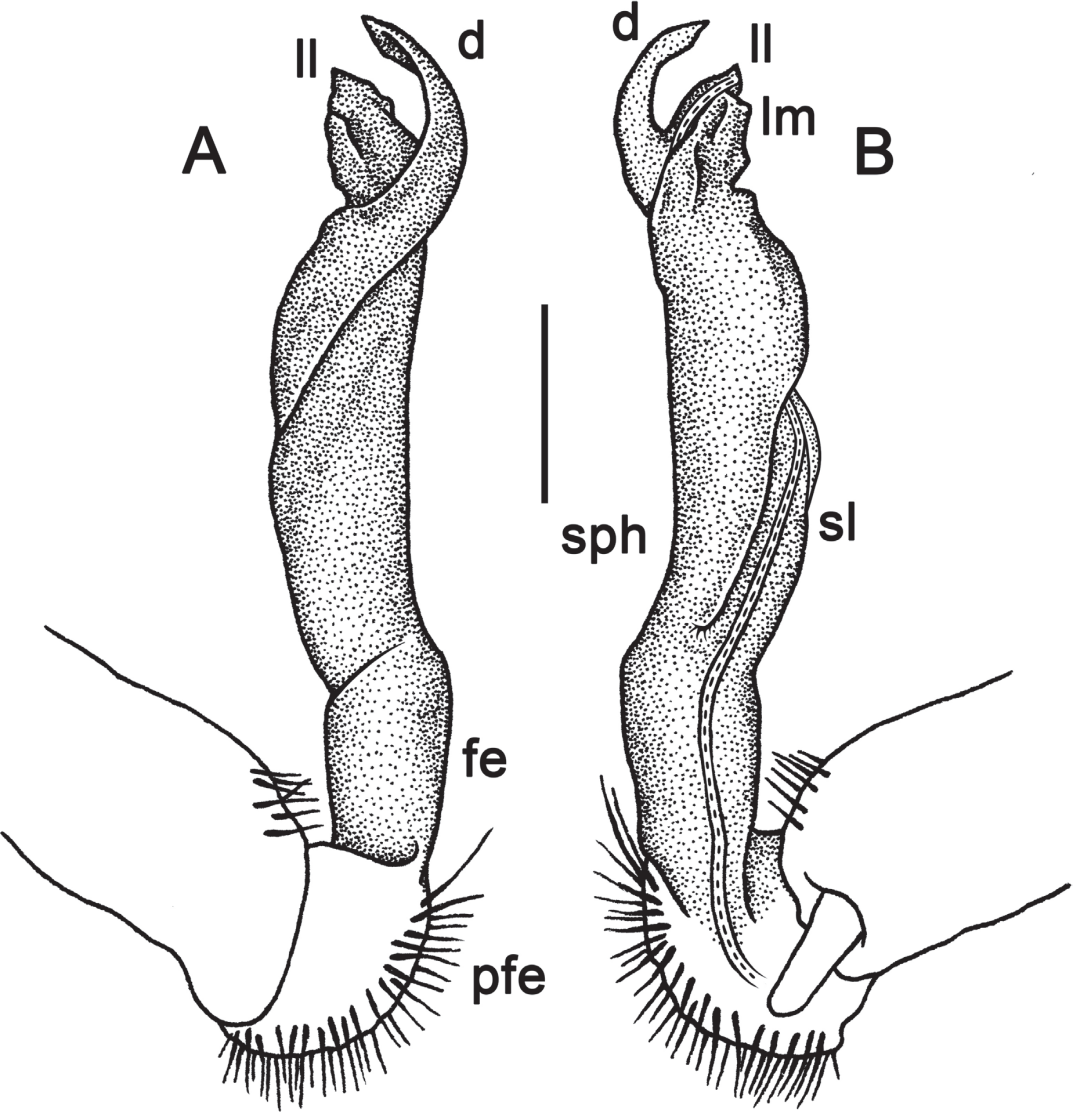
*Touranellachenla* sp. nov., ♂ holotype, left gonopod **A, B** lateral, mesal, suboral, and subcaudal views, respectively. Scale bars: 0.2 mm.

The new species seems to be particularly similar to *T.champasak* from Bolaven Plateau, Laos, especially in gonopodal structure. Both species share a strongly reduced gonopodal femorite devoid of a femoral process and a distally slightly twisted solenophore (Figs 5, 6). However, the new species differs from *T.champasak* by having a long, acute process d located around the mid-length of a more clearly erect and thicker solenophore (Figs 5, 6). Additionally, pleurosternal carinae are complete crests with sharp caudal denticles extending beyond the rear tergal margin only on rings 2 and 3, reduced to a rounded caudal crest on rings 4 and 5, and absent thereafter (Fig. 4C, E) (vs absent on rings 18–19). Moreover, ♂ tarsal brushes are present until ring 8 (vs until ring 16).

#### Description.

Length 15.2 mm, width of midbody pro- and metazona 1.1 and 1.3 mm, respectively (♂). Coloration of live animals dark brown (Fig. 4A); legs pale orange, venter and a few basal podomeres pale brown to yellow brown. Coloration in alcohol after 11 years of preservation faded to pale brown; antennae and epiproct pale brown to pallid, venter and podomeres pallid (Fig. 4B–J).

Clypeolabral region and vertex sparsely setose, epicranial suture distinct. Antennae long (Fig. 4A), reaching until ring 5 when stretched dorsally. In width, ring 4 < 3 < 2 < collum < ring 5 < 6 < 7 < head < 8–17, thereafter body gently and gradually tapering towards telson. Collum with three transverse rows of setae: 3+3 anterior, 2+2 intermediate, and 3+3 posterior; caudal corner very narrowly rounded, slightly declined ventrally, not produced past rear tergal margin (Fig. 4A–C).

Tegument smooth and shining, prozona finely shagreened, metaterga nearly smooth, faintly rugulose and leathery (Fig. 4B–F, H). Postcollum metaterga each with two transverse rows of setae: 2+2 anterior and 2+2 posterior, traceable at least as insertion points when setae broken off. Tergal setae simple, slender, short, ~ 1/3 metatergal length (Fig. 4F). Axial line well visible on metazona, traceable also on prozona.

Paraterga weak (Fig. 4A–F, H), lying at ~ 1/2 midbody height, slightly upturned, anterior edge rounded and narrowly bordered; caudal corner very narrowly rounded on rings 2–4, not produced past rear tergal margin; almost missing on following rings; pore-bearing rings with evident lateral bulges (Fig. 4B, D). Ozopores evident, lateral, each lying in an ovoid groove at ~ 1/3 metatergal length in front of posterior edge of metaterga (Fig. 4C, E, H). Transverse metatergal sulci complete on rings 5–16, incomplete on ring 17, very narrow, shallow, not reaching the bases of paraterga, at most faintly beaded at bottom (Fig. 4B–F, H). Stricture between pro- and metazona wide, deep, clearly ribbed at bottom down to base of paraterga (Fig. 4B–F, H). Pleurosternal carinae complete crests with a very sharp median tooth on rings 2 and 3, reduced to a rounded caudal crest on rings 4 and 5, thereafter missing (Fig. 4C, E, H).

Epiproct (Fig. 4F–H) conical, flattened dorsoventrally, with two small, rounded, apical papillae; tip subtruncate; lateral pre-apical papillae very small, lying close to tip. Hypoproct roundly subtriangular, setigerous knobs at caudal edge small and well-separated (Fig. 4G).

Sterna sparsely setose, without modifications; an entire, high, rounded, linguiform, setose sternal lobe between ♂ coxae 4 (Fig. 4I, J). Legs long, midbody legs 1.2–1.4 × as long as body height, prefemora without modifications, ♂ tarsal brushes present until ring 8.

Gonopods simple, slim and suberect (Figs 5, 6). Coxite slightly curved caudad, densely setose distodorsally (Figs 5A, B, 6A, B, D). Prefemoral part (= prefemorite, pfe) as usual, densely setose, ~ 3.5 × shorter than acropodite (femoral + postfemoral parts) (Figs 5A, B, 6A–C). Femorite (fe) short, with a strong, long, flagelliform solenomere (sl) twisted distad and with a strong, oblique, lateral sulcus demarcating a postfemoral part (Figs 5A, B, 6A–C). Solenophore (sph) long, slightly twisted distad, sheathing most of solenomere (sl), slightly curved caudad (Figs 5A, B, 6A–D). Solenophore consisting of a well-developed lamina lateralis (ll) and a smaller lamina medialis (lm) (Figs 5B, 6B, C). Tip of lamina medialis subtruncate (Figs 5B, 6B), tip of lamina lateralis subrectangular with a long, slender, curved, nearly pointed process d (d), this rising distal to lamina lateralis (Figs 5A, B, 6A–D). Both supporting a long and flagelliform solenomere.

#### Distribution.

Known only from the type locality, apparently endemic to the southern part of Laos.

#### Etymology.

The species epithet *chenla* refers to the ancient kingdom of Chenla, a powerful kingdom that thrived from approximately 550 to 802 AD across the present-day territories of Thailand, Laos, Cambodia, and Vietnam. The naming also reflects the historical connection between the Chenla era and the construction of the Vat Phou temple complex in Champasak Province, Laos, where the species was discovered (Wikipedia 2025a, b). This new species so named pays tribute to the rich cultural and historical heritage of the region.

#### Remarks.

The newly described species was discovered in the vicinity of Vat Phou, Champasak Province, Laos. This ruined Khmer Hindu temple complex, recognized as a UNESCO World Heritage Site, is situated amidst the lush landscapes of Southeast Asia, providing an intriguing backdrop for biodiversity exploration.

### ﻿ Touranella srisonchaii

Taxon classificationAnimaliaPolydesmidaParadoxosomatidae

Likhitrakarn sp. nov.

6075D95B-DFBC-598A-BF27-C5A3BAC7785F

https://zoobank.org/F56EDDFC-22F5-4E98-B4A0-D4EE86152E1F

Figs 7 , 8 , 9

#### Type material.

***Holotype*** • ♂ (CUMZ-PD0032), Laos, Champasak Province, Paksong District, Phu Thevada Hotel, 106 m, 15°10'50.4"N, 106°14'20.17"E, 24.2.2017, leg. R. Srisonchai.

***Paratypes***: • 25 ♂, 42 ♀ (CUMZ-PD0032), same locality, together with holotype, leg. R. Srisonchai.

#### Diagnosis.

**Figure 7.**
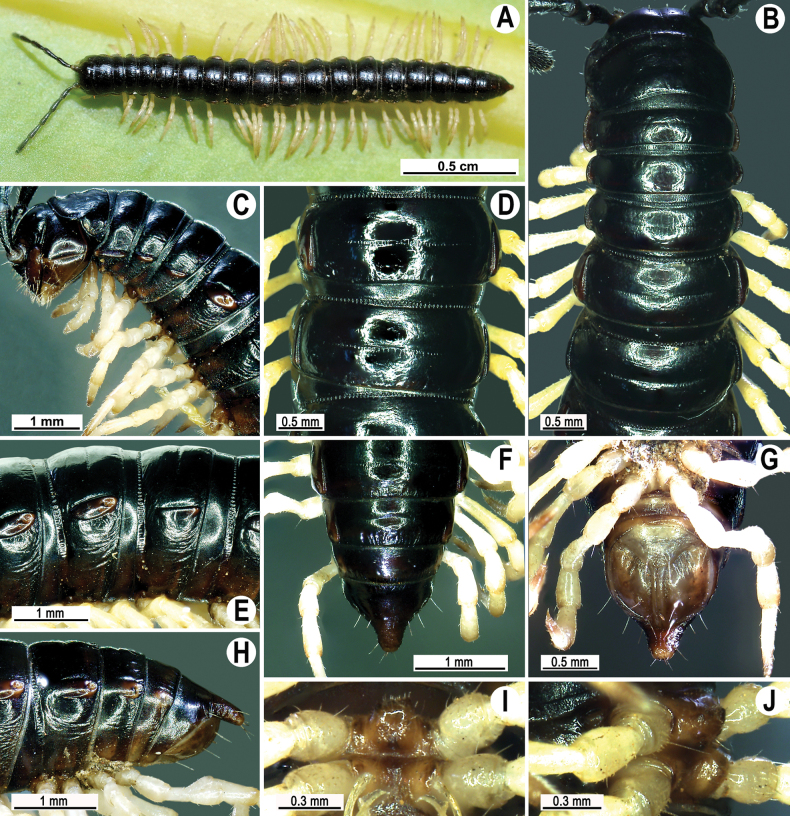
*Touranellasrisonchaii* sp. nov., ♂ holotype **A** habitus, live coloration **B, C** anterior part of body, dorsal and lateral views, respectively **D, E** rings 10 and 11, dorsal and lateral views, respectively **F–H** posterior part of body, dorsal, ventral, and lateral views, respectively **I, J** sternal cones between coxae 4, subcaudal and sublateral views, respectively.

**Figure 8.**
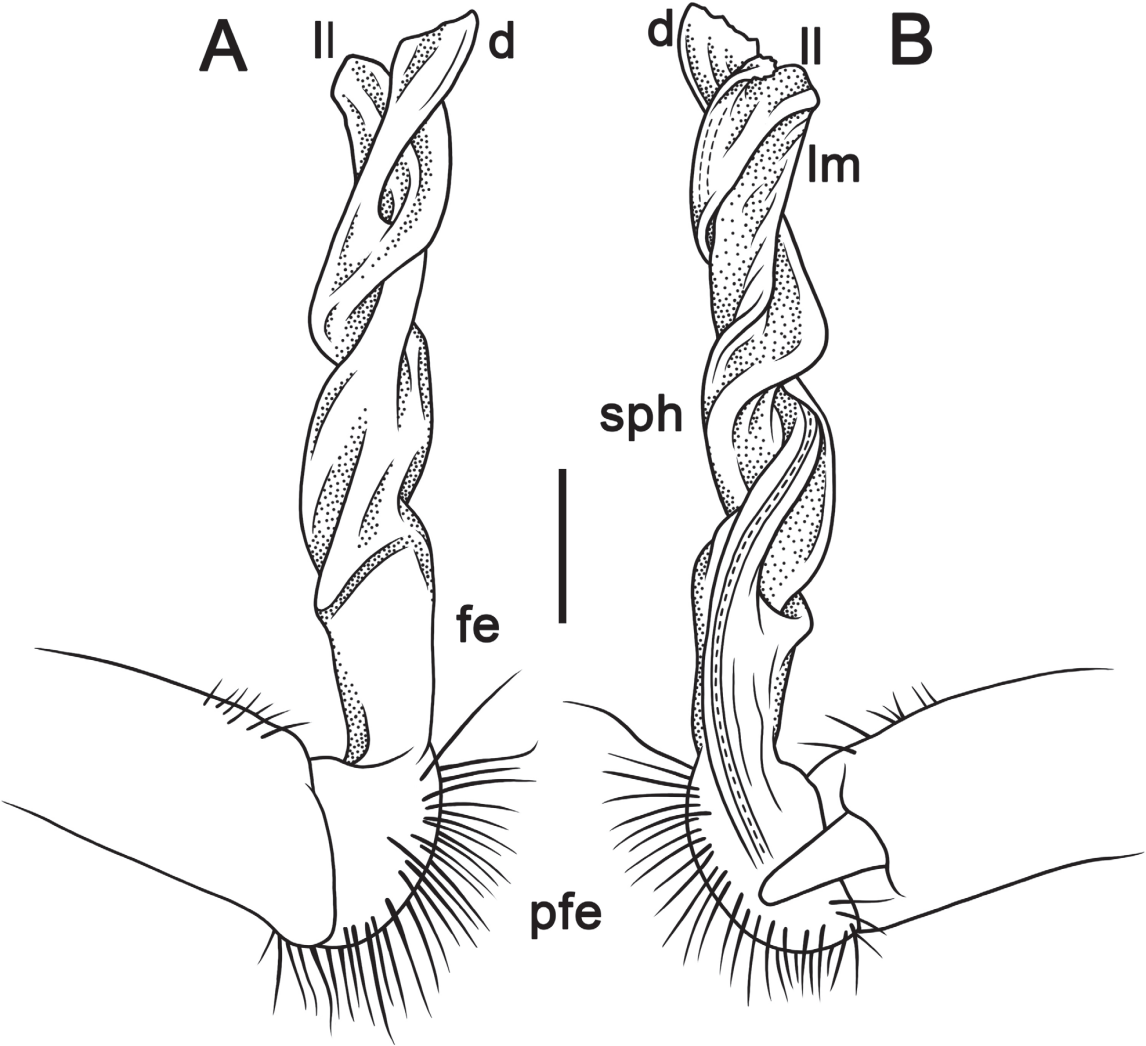
*Touranellasrisonchaii* sp. nov., ♂ holotype **A, B** right gonopod, lateral and mesal views, respectively. Abbreviations: d = process d, fe = femoral part, ll = lamina lateralis, lm = lamina medialis, pfe = prefemoral part, sl = solenomere, sph = solenophore. Scale bars: 0.2 mm.

**Figure 9.**
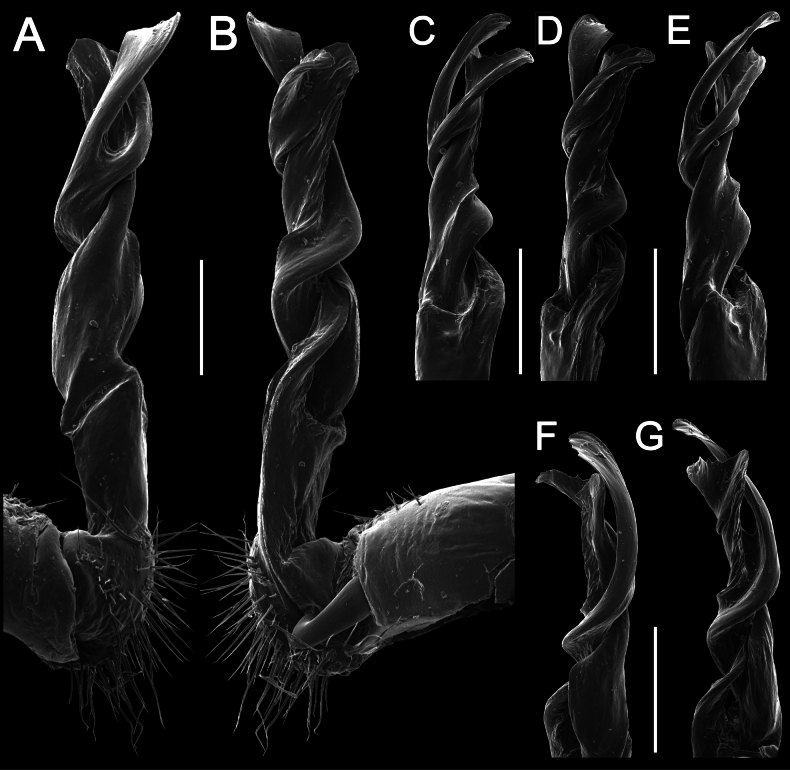
*Touranellasrisonchaii* sp. nov., ♂ holotype, right gonopod **A, B** lateral and mesal views, respectively **C–G** distal part, submesal, subcaudal, suboral, sublateral, and subcaudal views, respectively. Scale bars: 0.2 mm.

Morphologically, in gonopodal structure this new species seems to be especially similar to both *T.chenla* sp. nov. and *T.champasak*, but it is distinguished by the uniform black coloration lacking a cingulate pattern (Fig. 7A–H). It differs from *T.champasak* by having process d at the mid-length of the solenophore (Figs 8, 9) (vs absent); a more erect and thicker solenophore (Figs 8, 9) (vs slender and curved). *T.srisonchaii* sp. nov. differs from *T.chenla* sp. nov. in having ♂ tarsal brushes present until ring 8 (vs until ring 14) and a twisted solenophore with a laminate and subtruncate process d (Figs 8, 9) (vs a suberect gonopodal solenophore with a slender and acute process d, Figs 5, 6).

#### Description.

Length 14.8–21.0 (♂) or 14.6–19.8 mm (♀), width of midbody pro- and metazona 1.5–2.3 and 1.8–2.2 mm (♂) or 1.8–2.4 and 2.2–2.6 mm (♀), respectively. Coloration of live animals mostly dark or blackish (Fig. 7A); head, antennae, paraterga, and epiproct slightly lighter; venter dark brown, legs pale yellowish. Coloration in alcohol after seven years of preservation faded to dark brown or blackish; head, antennae, paraterga, and epiproct dark brown to pale brown; venter pale brown; legs pale yellowish to pallid (Fig. 7B–J).

Clypeolabral region and vertex sparsely setose, epicranial suture distinct. Antennae moderately long (Fig. 7A, B), reaching until body ring 4 (♂, ♀) when stretched dorsally. Head in width < collum < ring 3 < 4 < 2 < 5–16 (♂, ♀); thereafter body gently and gradually tapering towards telson. Collum with three transverse rows of setae: 3+3 anterior, 2+2 intermediate, and 4+4 posterior; caudal corner very narrowly rounded, slightly declined ventrally, not produced past rear tergal margin (Fig. 7B, C).

Tegument smooth and shining, prozona finely shagreened, metaterga finely rugulose (Fig. 7A–F, H); surface below paraterga finely microgranulate (Fig. 7C, E, F). Postcollum metaterga each with two transverse rows of setae: 3+3 anterior and 4+4 posterior, nearly always abraded, but still traceable as insertion points. Tergal setae simple, slender, short, ~ 1/5 metatergal length. Axial line well visible on metazona, traceable also on prozona.

Paraterga moderately developed (Fig. 7A–F, H), slightly upturned, lying at ~ 1/2 midbody height. Paraterga 2 subhorizontal, broad in dorsal view, thin in lateral view; shoulders well-developed, slightly rounded and oblique laterally; caudal tip rounded, slightly produced past rear tergal margin (Fig. 7B, C). Following rings with evident lateral bulges, not produced past rear tergal margin, broader on pore-bearing rings (Fig. 7B, D, F). Ozopores evident, lateral, each lying in an ovoid groove at ~ 1/3 metatergal length in front of posterior edge of metaterga (Fig. 7C, E, H).

Transverse metatergal sulci usually distinct (Fig. 7B–F, H), incomplete on rings 4 and 19, complete on metaterga 5–17 (♂, ♀), shallow, not reaching the bases of paraterga, at most faintly beaded at bottom. Stricture between pro- and metazona narrow, beaded at bottom down to base of paraterga (Fig. 7B–E). Pleurosternal carinae complete crests with a sharp caudal tooth on ring 2, reduced to a rounded caudal crest on rings 3 and 4, thereafter missing (♂, ♀) (Fig. 7C, E, H).

Epiproct (Fig. 7F–H) conical, flattened dorsoventrally, with two small, rounded, apical papillae; tip rounded; lateral pre-apical papillae very small, lying close to tip. Hypoproct roundly subtriangular, setigerous knobs at caudal edge small and well-separated (Fig. 7G).

Sterna sparsely setose, without modifications (Fig. 7G); an entire, rather short, rounded, linguiform, setose, sternal lobe between ♂ coxae 4 (Fig. 7I, J). A paramedian pair of evident tubercles in front of gonopodal aperture. Legs moderately long and slender, midbody ones ~ 1.1–1.3 (♂) or 0.9–1.1 × (♀) as long as body height, prefemora without modifications, ♂ tarsal brushes present until ring 14.

Gonopods relatively simple and suberect (Figs 8, 9). Coxite slightly curved caudally, rather densely setose distodorsally (Figs 8A, B, 9A, B). Prefemoral part (pfe) densely setose, ~ 1/3 as long as acropodite (femoral + postfemoral parts) (Figs 8A, B, 9A, B). Femorite (fe) rather short, with a medial, strong, long, flagelliform solenomere (sl), strongly twisted distally, and with an oblique lateral sulcus defining a postfemoral part (Figs 8A, B, 9A, B). Solenophore (sph) long, stout, suberect, with a clear lateral shoulder, sheathing most of solenomere (sl) (Figs 8A, B, 9A–D). Lamina lateralis (ll) well-developed, strongly twisted, and lamina medialis (lm) suberect (Figs 8B, 9D, E). Tip of lamina lateralis a broad, expanded, apical lamina with three small denticles (Figs 8A, B, 9A–G), at halfway bearing a large, long, slightly curved, subtruncate tip process d, this rising distal to lamina lateralis (Figs 8A, B, 9A–G).

#### Distribution.

Known only form the type locality, apparently endemic to the southern part of Laos.

#### Etymology.

To honor Dr. Ruttapon Srisonchai, diplopodologist at the Faculty of Science of Khon Kaen University, who has not only contributed to the study of millipede taxonomy in Thailand, but also collected the type series of this new species.

#### Remarks.

The new species was discovered near Phu Thevada Hotel in the evening following a rainfall, after a full day of collecting. The area surrounding the hotel is a small pine forest situated on a low hill. The rainfall prompted millipedes and land snails to emerge on the ground and tree trunks, facilitating the collection of a significant number of samples. In total, 67 specimens were taken, revealing a male to female ratio of 1:1.68.

### ﻿ Touranella jaegeri

Taxon classificationAnimaliaPolydesmidaParadoxosomatidae

Likhitrakarn sp. nov.

2763A5D6-3EF9-5FD1-A24F-10D263463331

https://zoobank.org/B0940D80-C5CC-49CF-9AAD-335236EE643D

Figs 10 , 11 , 12

#### Type material.

***Holotype*** • ♂ (SMF-SM-01), Laos, Bolikhamsay Province, Lak Sao, Tham Mankhone, 501 m, 18°13'16.1"N, 104°48'45.9"E, 21.7.2016, leg. P. Jäger. ***Paratypes***: • 1 ♂ (SMF-SM-01), same locality, together with holotype.

#### Diagnosis.

The new species closely resembles both *T.chenla* sp. nov. and *T.champasak*, especially in its moniliform body with significantly reduced paraterga (Fig. 4A–H, 7A–H). However, it differs from *T.chenla* sp. nov. by possessing a broader and better expanded apical process d, which arises proximal to the lamina lateralis (Figs 11, 12) (vs a slender, long, curved, apical process d that rises distal to the lamina lateralis (Figs 5, 6)), coupled with a larger size, measuring 21.4–22.6 mm long (vs smaller, 15.2 mm long). In comparison to *T.champasak*, this species has pleurosternal carinae with complete crests and sharp caudal teeth on rings 2–4, and absent from ring 5 (vs present until ring 17 and absent thereafter). ♂ legs are 1.3–1.5 × as long as midbody height (vs longer, 1.7–1.8×), and ♂ tarsal brushes present until ring 10 (vs until ring 16).

#### Description.

**Figure 10.**
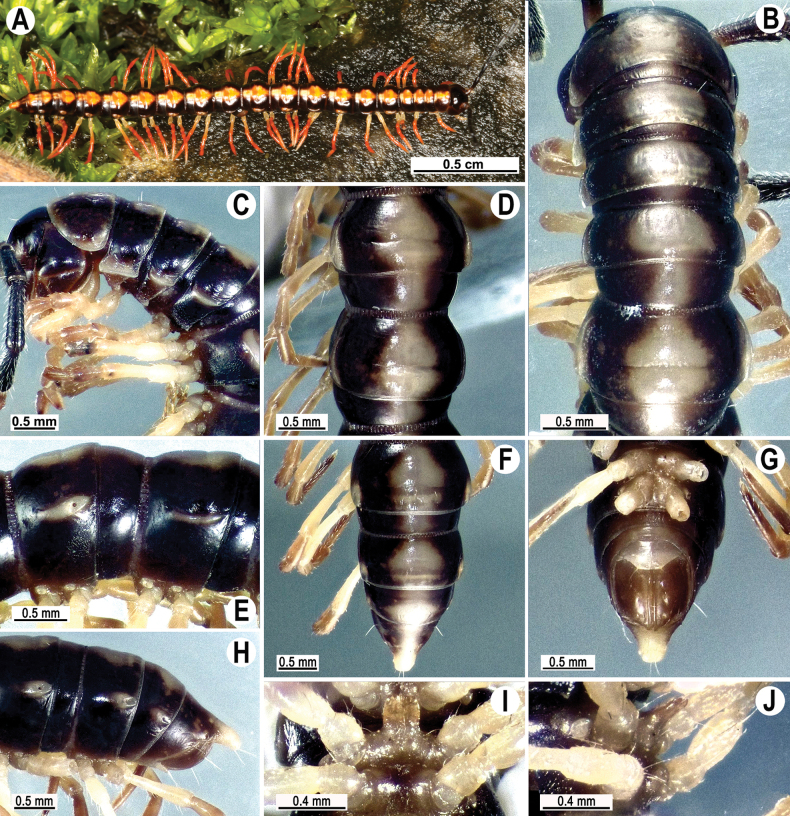
*Touranellajaegeri* sp. nov., ♂ holotype **A** habitus, live coloration **B, C** anterior part of body, dorsal and lateral views, respectively **D, E** rings 10 and 11, dorsal and lateral views, respectively **F–H** posterior part of body, dorsal, ventral, and lateral views, respectively **I, J** sternal cones between coxae 4, subcaudal and sublateral views, respectively.

Length 21.4–22.6 (♂), width of midbody pro- and metazona 2.6–2.8 and 3.2–3.3 mm (♂), respectively. Coloration of live animals dark brown-orange (Fig. 10A), with a contrasting longitudinal pale yellow-orange stripe, this being narrow on prozona, but characteristically bead-shaped and broadened on metazona; paraterga, epiproct, and venter pale orange to pale yellow; head and antennae dark brown; legs contrasting pale orange, a few basal podomeres pale yellow to pallid. Coloration in alcohol after eight years of preservation faded to dark brown; antennae dark brown and increasingly faded distally; paraterga, venter, and epiproct pale brown to pallid; and podomeres pallid, basal podomeres of legs pallid, increasingly dark brown distally (Fig. 10B–J).

**Figure 11.**
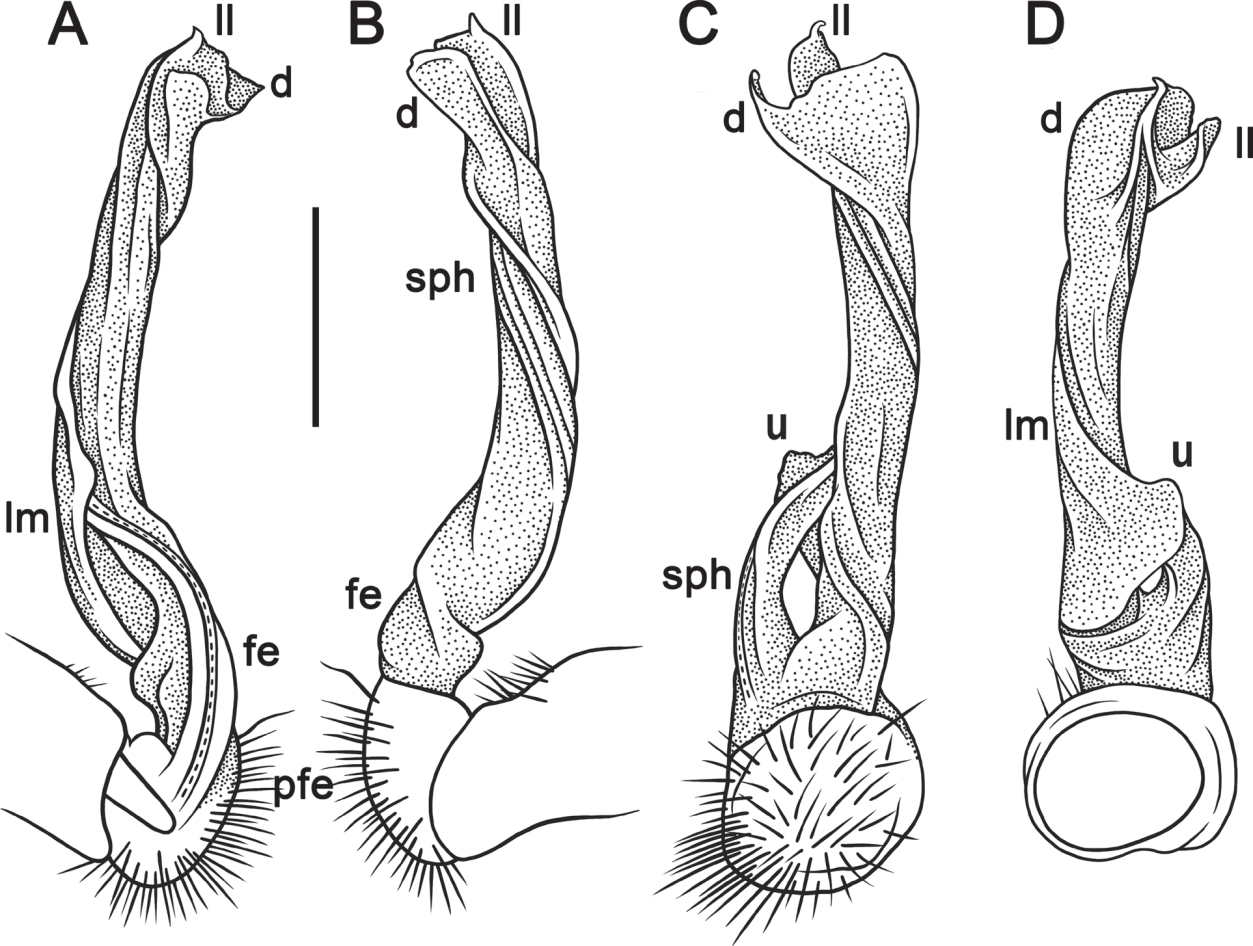
*Touranellajaegeri* sp. nov., ♂ holotype, left gonopod **A–D** mesal, lateral, suboral, and subcaudal views, respectively. Abbreviations: d = process d, fe = femoral part, ll = lamina lateralis, lm = lamina medialis, pfe = prefemoral part, sph = solenophore, u = shoulder u. Scale bar: 0.2 mm.

**Figure 12.**
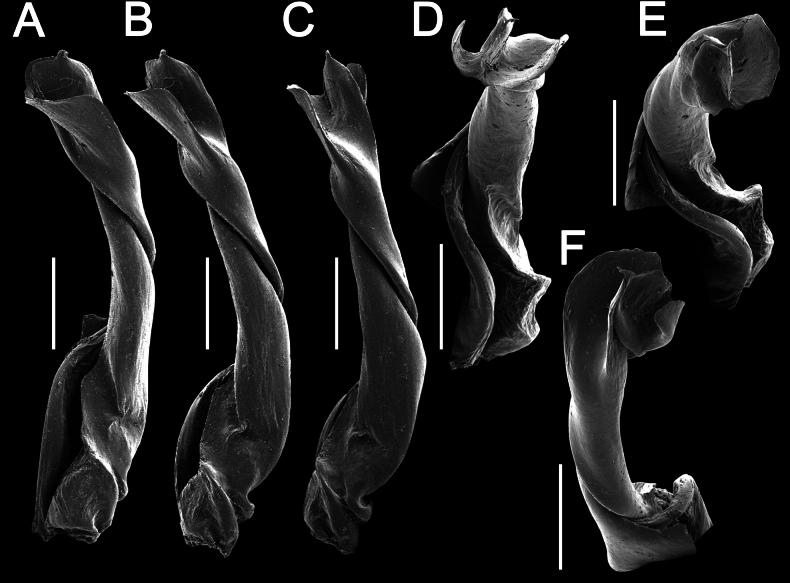
*Touranellajaegeri* sp. nov., ♂ holotype, left gonopod, acropodite part **A–F** sublateral, lateral, sublateral, suboral, submesal, and subcaudal views, respectively. Scale bars: 0.2 mm.

Clypeolabral region and vertex sparsely setose, epicranial suture distinct. Antennae long, reaching until ring 5 when stretched dorsally (Fig. 10A). In width, ring 3 = 4 < collum < ring 2 < 5 < 6–17 (♂); thereafter body gently and gradually tapering towards telson. Collum with three transverse rows of setae: 3+3 anterior, 2+2 intermediate, and 4+4 posterior; caudal corner very narrowly rounded, paraterga slightly declined ventrally, not produced past rear tergal margin (Fig. 10A, B).

Tegument smooth and shining, prozona finely shagreened, metaterga nearly smooth, faintly rugulose and leathery (Fig. 10B–F, H) Postcollum metaterga each with two transverse rows of setae: 2+2 in anterior and 3+3 in posterior row, the latter traceable as insertion points when setae broken off. Tergal setae simple, short, slender, ~ 1/5 metatergal length. Axial line visible on metazona, traceable also on prozona. Paraterga weak (Fig. 10A–F, H), slightly upturned posteriorly, lying at ~ 1/2 midbody height, lateral edge without incisions. Paraterga 2 subhorizontal, broad in dorsal view, thin in lateral view, anterior edge well-developed, slightly rounded and oblique laterally, caudal tip rounded, slightly produced past rear tergal margin (Fig. 10B, C).

Paraterga 3 and 4 with evident lateral bulges, caudal corner very broadly rounded, not produced past rear tergal margin (Fig. 10B, C). Following rings with small lateral bulges, not produced past rear tergal margin, broader on pore-bearing rings (Fig. 10B, D, F). Calluses on paraterga delimited by a sulcus both dorsally and ventrally. Ozopores evident, lateral, each lying in an ovoid groove at ~ 1/3 metatergal length in front of posterior edge of metaterga (Fig. 10E, H). Transverse metatergal sulci usually distinct (Fig. 10B–F, H), incomplete on ring 17, complete on rings 5–16, shallow, not reaching the bases of paraterga, at most faintly beaded at bottom. Stricture between pro- and metazona wide and rather deep, ribbed at bottom down to base of paraterga (Fig. 10B–E). Pleurosternal carinae complete crests with sharp caudal teeth on rings 2–4, thereafter missing (Fig. 10C, E, H).

Epiproct (Fig. 10F–H) conical, flattened dorsoventrally, tip subtruncate, subapical lateral papillae small, but visible, lying close to tip. Hypoproct roundly subtrapeziform, small setigerous knobs at caudal edge small and well-separated (Fig. 10G).

Sterna sparsely setose, without modifications (Fig. 10G); an entire, high, rounded, linguiform, setose, sternal lobe between ♂ coxae 4 (Fig. 10I, J). A paramedian pair of evident tubercles in front of gonopodal aperture. Legs long and slender, midbody ones ~ 1.3–1.5 as long as body height; prefemora without modifications; ♂ tarsal brushes present until ring 10 (♂).

Gonopods relatively simple and suberect (Figs 11, 12). Coxite slightly curved caudally, rather densely setose distodorsally (Fig. 11A, B). Prefemoral part (pfe) densely setose, ~ 1/4 as long as acropodite (femoral + postfemoral parts) (Fig. 11A–C). Femorite (fe) strongly reduced, without femoral processes, bearing a medial, strong, long, flagelliform solenomere (sl), twisted distally, and with an oblique lateral sulcus defining a postfemoral part (Fig. 11A, B). Solenophore (sph) long, rather slender, slightly curved caudally, sheathing most of solenomere (sl), directed caudally, with a lateral shoulder (u) at base (Figs 11C, D, 12A, D, E). Solenophore consisting of a well-developed, strongly twisted lamina lateralis (ll) and a smaller, slightly twisted lamina medialis (lm) (Fig. 11A–D). Tip of the lamina lateralis (ll) split into two broad, expanded apical laminae, a median lamina with a small denticle, and halfway bearing a broad, expanded apical lamina (Figs 11A, D, 12C–F), subtruncate tip process d rising proximal to lamina lateralis (Fig. 11A, B).

#### Distribution.

Known only from the type locality, apparently endemic to the central part of Laos.

#### Etymology.

To honor Dr. Peter Jäger, a renowned arachnologist from the Senckenberg Research Institute in Frankfurt, Germany, who collected the type specimens of this species.

#### Remarks.

The new species was discovered during a survey undertaken as part of the Laos Biodiversity Survey project.

##### ﻿Key to the currently known species of *Touranella* (chiefly based on ♂), modified after Nguyen et al. (2023)

**Table:** 

1	Most metaterga granulate-tuberculate or densely hirsute (Fig. 2A–G, J–L)	**2**
–	Most metaterga smooth, with only two or three transverse rows of setae on each ring (Figs 4A–H, 7A–H, 10A–H)	**6**
2	Gonopodal femoral process absent (Figs 5, 6, 8, 9, 11, 12)	**3**
–	Gonopodal femoral process present (Fig. 3)	**4**
3	Postcollum metaterga clothed with long hairs placed inside minute pores/knobs. Gonopodal solenophore with a vestigial parabasal lobe, a distinct, acuminate, apical uncus, and a couple of characteristic subapical outgrowths	***T.trichosa***
–	Postcollum metaterga with six transverse regular rows of tergal setae borne on small tubercles/knobs per ring. Gonopodal solenophore with neither a basal process nor an apical uncus	***T.hirsuta***
4	Gonopodal femorite carrying three processes	***T.pilosa***
–	Gonopodal femorite carrying only a single process	**5**
5	Femoral process long. Basal lateral shoulder of solenophore larger, well developed. Nepal	***T.himalayaensis***
–	Femoral process short. Basal lateral shoulder of solenophore small and less strongly developed (Fig. 3). Vietnam	***T.gracilis***
6	Body larger: 25 mm long, 1.6 and 2.0 mm wide on pro- and metaterga, respectively. Gonopodal femoral process present	***T.moniliformis***
–	Body smaller. Gonopodal femoral process absent	**7**
7	Body smaller: 9–14 mm long. Antennae shorter, *in situ* extending only past body ring 3 when stretched dorsally. Midbody paraterga moderately developed, each with one or two lateral setigerous knobs and a pointed caudal corner. Pleurosternal carinae either absent or present at most until ring 4	**8**
–	Body larger: > 14.8 mm long. Antennae longer, *in situ* reaching at least body ring 4 when stretched dorsally. Midbody paraterga weakly developed, with lateral bulges, caudal corner very broadly rounded (Figs 4D, 7D, 10D). Pleurosternal carinae present at least until ring 4	**10**
8	Pleurosternal carinae absent. ♂ tarsal brushes present on legs l–5(6). Gonopodal solenophore long and slender, with a basal lateral shoulder	***T.peculiaris***
–	Pleurosternal carinae present. ♂ tarsal brushes entirely absent. Gonopodal solenophore stout, without basal shoulder	**9**
9	Body larger: 13.5 mm long. Most of postcollum metaterga with four transverse rows of 5–6 setae per row. Pleurosternal carinae present on rings 2–4. ♂ legs longer, 2.5–3.0 × as long as midbody height. Gonopodal solenophore elongate and ribbon-shaped	***T.logunovi***
–	Body smaller: 9–10 mm long. Postcollum metaterga with three transverse rows of 3(4)–3(4) setae per row. Pleurosternal carinae present only on ring 2. ♂ legs shorter, 1.2–1.3 × as long as midbody height. Gonopodal solenophore rod-shaped	***T.cattiensis***
10	Coloration uniformly black, without cingulate pattern (Fig. 7A–H). Body rather juliform (Fig. 7A–H). Antennae shorter, *in situ* reaching until body ring 4 when stretched dorsally. Gonopodal solenophore with an evident lateral shoulder (Figs 8, 9)	***T.srisonchaii* sp. nov.**
–	Coloration brownish, with a cingulate pattern (Figs 4A–H, 10A–H). Body submoniliform (Figs 4A–H, 10A–H). Antennae longer, in situ reaching until body ring 5 when stretched dorsally. Gonopodal solenophore without evident lateral shoulder (Figs 5, 6, 11, 12)	**11**
11	Body smaller: 15.2 mm long, 1.1 and 1.3 mm wide on pro- and metaterga, respectively. Coloration brownish, without axial band (Fig. 4A–H). ♂ tarsal brushes present until ring 8. Gonopodal solenophore twisted, with a laminate and subtruncate tip process d (Figs 5, 6)	***T.chenla* sp. nov.**
–	Body larger: 21.4–22.6 mm long, 1.5–2.6 and 1.9–3.3 mm wide on pro- and metaterga, respectively. Coloration dark brown, with a contrasting pale axial band running from collum to telson (Fig. 10A–H). ♂ tarsal brushes present at least until ring 10. Gonopodal solenophore suberect, with a slender and acute process d (Figs 10, 11)	**12**
12	Body smaller: 1.5–1.9 mm wide. Sternal lobe between ♂ coxae 4 trapeziform. ♂ legs longer, 1.7–1.8 × as long as midbody height. Tarsal brushes present until legs of ♂ ring 16. Pleurosternal carinae complete crests with sharp caudal teeth on rings 2–4, present until ring 18 or 19	***T.champasak***
–	Body broader: 2.6–3.3 mm wide. Sternal lobe between ♂ coxae 4 high and linguiform (Fig. 10I, J). ♂ legs shorter, 1.3–1.5 × as long as midbody height. Tarsal brushes present until legs of ♂ ring 10. Pleurosternal carinae complete crests and sharp caudal teeth on rings 2–4, but missing after ring 5 (Fig. 10C)	***T.jaegeri* sp. nov.**

## ﻿Discussion and conclusions

Of a total of 13 species of *Touranella* known now, Vietnam supports as many as seven, followed by Laos (four species), and Nepal (two species). This genus of Alogolykini is distinguished from other contribal genera by a strong, suberect, and rod-shaped solenomere (vs a thin, flagelliform solenomere in the tribe Polydrepanini) (Likhitrakarn et al. 2013; Golovatch et al. 2021; Nguyen et al. 2023). Both tribes are the only members of the Alogolykinae that differ only by the absence in Polydrepanini of a clear-cut division of the solenophore or solenophore-like structure near/around the solenomere into a membranous lamina medialis and/or a similarly membranous lamina lateralis, also in Polydrepanini often being coupled with a twisted, helicoid course of the seminal groove. In addition, the gonopodal femorite in Alogolykinae is often strongly abbreviated, while many species show adenostyles on the first ♂ femora (Likhitrakarn et al. 2013). The tribe Alogolykini currently comprises seven genera that are basically confined to the Oriental Region (Golovatch et al. 2021; Nguyen et al. 2023).

The genus *Touranella* is quite widespread, ranging from the Himalayas of Nepal in the west to Vietnam in Indochina in the east (Fig. 1), but virtually all of its species are narrow endemics mostly known from a single locality. Their habitat preferences and ecological amplitudes are quite diverse, but their dwelling in woodlands is manifest. In the northwestern part of the range, both *T.himalayaensis* and *T.pilosa* have been recorded from Nepal at 2,300–2,800 m a.s.l., indicating their adaptations to mid-montane environments in the Himalayan ecosystem (Golovatch and Martens 2018). All other presently known *Touranella* species are distributed across the central and southern regions of the Annamite Range. The remarkable gap between the Himalayas and the Annamite Range suggests there are more undiscovered species to be revealed (Fig. 1). In Vietnam alone, the seven already reported species indicate that further explorations in mainland Indochina may well reveal additional congeners.

Sympatric occurrences within the genus have also been observed. For example, three species of *Touranella*: *T.hirsuta*, *T.peculiaris* and *T.logunovi*, have been collected from the Bidoup – Nui Ba Nature Park in Lam Dong Province, Vietnam. This suggests potential overlaps in distribution, which could indicate ecological partitioning or temporal shifts in activity. The three species in the same area can be told apart by their very distinct morphologies. For instance, *T.hirsuta* shows a prominent granulate-tuberculate surface of the metaterga, while *T.peculiaris* and *T.logunovi* both have smooth bodies; nevertheless *T.logunovi* has a larger body, longer legs and denser setose metaterga compared to *T.peculiaris*. Similarly, *T.cattiensis* and *T.moniliformis* have both been discovered in the Cat Tien National Park in Dong Nai Province, Vietnam, albeit at different times. This suggests that seasonal variations may have influenced their discoveries. Likewise, *T.cattiensis* is distinguished by its small body size (9–10 mm long) and the absence of a gonopodal femoral process, whereas *T.moniliformis* is significantly larger (25 mm longer) and it shows a gonopodal femoral process. Similar patterns of seasonal activity have also been observed in some other Southeast Asian groups of Paradoxosomatidae. For example, the genus *Tylopus* Jeekel, 1968 has been reported to occur sympatrically in such larger montane areas as Doi Inthanon (10 species) and Doi Suthep (10 species) in Thailand (Likhitrakarn et al. 2016), as well as within the dragon millipede genus *Desmoxytes* Chamberlin, 1923 (Srisonchai et al. 2018).

The distribution pattern of *Touranella* suggests historical biogeographical processes, such as vicariant events and dispersal mechanisms, influencing current diversity. The presence of species both in mid-altitude and lowland regions points to a complex evolutionary history and potential ecological adaptations allowing different *Touranella* species to thrive in varied environments. Moreover, comprehensive surveys in underexplored regions such as Laos, Vietnam, Cambodia, and Thailand are essential to identify and describe yet undiscovered species, further enhancing our understanding of *Touranella* species diversity and their distributions.

## Supplementary Material

XML Treatment for Touranella

XML Treatment for Touranella gracilis

XML Treatment for Touranella himalayaensis

XML Treatment for Touranella peculiaris

XML Treatment for Touranella hirsuta

XML Treatment for Touranella cattiensis

XML Treatment for Touranella pilosa

XML Treatment for Touranella moniliformis

XML Treatment for Touranella trichosa

XML Treatment for Touranella champasak

XML Treatment for Touranella logunovi

XML Treatment for Touranella chenla

XML Treatment for Touranella srisonchaii

XML Treatment for Touranella jaegeri

## ﻿Additional information

### Author ORCIDs

Natdanai Likhitrakarn https//orcid.org/0000-0002-1306-317X

Sergei I. Golovatch https//orcid.org/0000-0001-7159-5484

Khamla Inkhavilay https//orcid.org/0009-0007-1060-0885

Somsak Panha https//orcid.org/0000-0002-4431-2458

Chirasak Sutcharit https//orcid.org/0000-0001-7670-9540

### Data availability

All of the data that support the findings of this study are available in the main text.

## References

[B1] Attems CW (1937) Myriapoda 3. Polydesmoidea I. Fam. Strongylosomidae. Das Tierreich 68: 1–300. 10.1515/9783111567099

[B2] Attems C (1938) Die von Dr. C. Dawydoff in französisch Indochina gesammelten Myriopoden. Mémoires du Muséum national d’histoire naturelle N.S. 6(2): 187–353.

[B3] American Veterinary Medical Association2020. AVMA Guidelines for the Euthanasia of Animals: 2020 Edition. https://www.avma.org/resources-tools/avma-policies/avma-guidelines-euthanasia-animals [Accessed 18 April 2025]

[B4] Enghoff H Golovatch SI Nguyen AD (2004) A review of the millipede fauna of Vietnam (Diplopoda). Arthropoda Selecta 13(1–2): 29–43.

[B5] Enghoff H Golovatch SI Short M Stoev PE Wesener T (2015) Diplopoda – taxonomic overview, In: Minelli A (Ed.) Treatise on Zoology – Anatomy, Taxonomy, Biology.The Myriapoda. Vol. 2. Brill, Leiden & Boston, 363–453.

[B6] Golovatch SI (1983) Millipedes (Diplopoda) of the fauna of Vietnam. In: Medvedev LN (Ed.) Fauna and ecology of the animals of Vietnam. Nauka, Moscow, 207 pp. [In Russian]

[B7] Golovatch SI (1994) Diplopoda from the Himalayas. Two new Alogolykini (Polydesmida: Paradoxosomatidae). Senckenbergiana biologica 73(1–2): 183–187

[B8] Golovatch SI (2009a) On several new or poorly-known Oriental Paradoxosomatidae (Diplopoda: Polydesmida), IX. Arthropoda Selecta 18(3/4): 119–124.

[B9] Golovatch SI (2009b) On several new or poorly-known Oriental Paradoxosomatidae (Diplopoda: Polydesmida), VIII. Arthropoda Selecta 18(1/2): 1–7. 10.15298/arthsel.19.3.02

[B10] Golovatch SI (2016) On several new or poorly-known Oriental Paradoxosomatidae (Diplopoda: Polydesmida), XIX. Arthropoda Selecta 25(2): 131–152. 10.15298/arthsel.25.2.01

[B11] Golovatch SI (2024) A new species and some new records of millipedes (Diplopoda) from southern Vietnam. Russian Entomological Journal 33(3): 407–413. 10.15298/rusentj.33.3.14

[B12] Golovatch SI Martens J (2018) Distribution, diversity patterns and faunogenesis of the millipedes (Diplopoda) of the Himalayas. In: Stoev P Edgecombe GD (Eds) Proceedings of the 17th International Congress of Myriapodology, Krabi, Thailand.ZooKeys 741: 3–34. 10.3897/zookeys.741.20041 PMC5904548 29706770

[B13] Golovatch SI Semenyuk II (2010) On several new or poorly-known Oriental Paradoxosomatidae (Diplopoda: Polydesmida), X. Arthropoda Selecta 19(3): 123–127. 10.15298/arthsel.19.3.02

[B14] Golovatch SI Semenyuk II (2018) On several new or poorly-known Oriental Paradoxosomatidae (Diplopoda: Polydesmida), XXIII. Arthropoda Selecta 27(1): 1–21. 10.15298/arthsel.27.1.01

[B15] Golovatch SI Tiunov AV Anichkin AE (2011) 4. Millipedes (Diplopoda). In: Tiunov AV (Ed.) Structure and functions of soil communities of a monsoon tropical forest (Cat Tien National Park, southern Vietnam).KMK Scientific Press, Moscow, 76–90. [In Russian, with an English abstract]

[B16] Golovatch SI Aswathy MD Bhagirathan U Sudhikumar AV (2021) Review of the millipede tribe Polydrepanini, with the description of a new species from Kerala state, southern India (Diplopoda, Polydesmida, Paradoxosomatidae, Alogolykinae). Zootaxa 5068(4): 485–516. 10.11646/zootaxa.5068.4.2 34810694

[B17] Hoffman RL (1963) A contribution to the knowledge of Asiatic strongylosomoid Diplopoda (Polydesmida: Strongylosomatidae). Annals and Magazine of Natural History (Series 13) 5: 577–593. 10.1080/00222936208651289

[B18] Hoffman RL (1980) Classification of the Diplopoda. Muséum d’histoire naturelle, Genève, 1–237.

[B19] Jeekel CAW (1968) On the classification and geographical distribution of the family Paradoxosomatidae (Diplopoda, Polydesmida). Thesis, University of Amsterdam, Rotterdam, 162 pp.

[B20] Likhitrakarn N Golovatch SI Panha S (2013) The millipede genus *Tetracentrosternus* Pocock, 1895 (Polydesmida, Paradoxosomatidae, Alogolykinae, Alogolykini), with a description of the first, new species from Thailand. ZooKeys 358: 1–10. 10.3897/zookeys.358.6582 PMC3867176 24363581

[B21] Likhitrakarn N Golovatch SI Panha S (2016) The millipede genus *Tylopus* Jeekel, 1968 (Diplopoda, Polydesmida, Paradoxosomatidae), with a key and descriptions of eight new species from Indochina. European Journal of Taxonomy 195: 1–47. 10.5852/ejt.2016.195

[B22] Nguyen AD Sierwald P (2013) A worldwide catalog of the family Paradoxosomatidae Daday, 1889 (Diplopoda: Polydesmida). Check List 9(6): 1132–1353. 10.15560/9.6.1132

[B23] Nguyen AD Sierwald P Ware S (2023) First record of the genus *Touranella* Attems, 1937 (Diplopoda, Polydesmida, Paradoxosomatidae) from Laos, with a description of a new species. ZooKeys 1145: 169–180. 10.3897/zookeys.1145.98704 37215403 PMC10193849

[B24] Semenyuk II Tiunov AV Golovatch SI (2011) Structure of mandibles in relation to trophic niche differentiation in a tropical millipede community. In: Mesibov R Short M (Eds) Proceedings of the 15th International Congress of Myriapodology, 18–22 July 2011, Brisbane, Australia.International Journal of Myriapodology 6: 37–49. 10.3897/ijm.6.2214

[B25] Srisonchai R Enghoff H Likhitrakarn N Panha S (2018) A revision of dragon millipedes I: genus *Desmoxytes* Chamberlin, 1923, with the description of eight new species (Diplopoda, Polydesmida, Paradoxosomatidae). ZooKeys 761: 1–177. 10.3897/zookeys.761.24214 PMC5988806 29875597

[B26] Wikipedia (2025a) Wikipedia the free encyclopedia: Chenla. https://en.wikipedia.org/wiki/ Chenla [Accessed 18 April 2025]

[B27] Wikipedia (2025b) Wikipedia the free encyclopedia: Vat_Phou. https://en.wikipedia.org/wiki/ Vat_Phou [Accessed 18 April 2025]

